# Global Association of the COVID-19 Pandemic With Pediatric Clinical Trial Publication

**DOI:** 10.1001/jamanetworkopen.2023.26313

**Published:** 2023-07-28

**Authors:** Sarah Grantham-Hill, Michael Eyre, Athimalaipet V. Ramanan, Neena Modi, Saskia N. de Wildt, Ming Lim

**Affiliations:** 1 Children’s Neurosciences, Evelina London Children’s Hospital at Guy’s and St Thomas’ National Health Service Foundation Trust, King’s Health Partners Academic Health Science Centre, London, United Kingdom; 2 School of Biomedical Engineering and Imaging Sciences, King’s College London, United Kingdom; 3 University Hospitals Bristol National Health Service Foundation Trust, Bristol, United Kingdom; 4 Translational Health Sciences, University of Bristol, Bristol, United Kingdom; 5 Chelsea and Westminster National Health Service Foundation Trust, London, United Kingdom; 6 Section of Neonatal Medicine, Department of Public Health and Primary Care, Imperial College London, London, United Kingdom; 7 Department of Pediatric and Neonatal Intensive Care, Erasmus MC Sophia Children’s, Hospital, Rotterdam, the Netherlands; 8 Department of Pharmacy, Division of Pharmacology and Toxicology, Radboud Institute for Health Sciences, Radbound University Medical Center, Nijmegen, the Netherlands; 9 Department of Women and Childrens Health, School of Life Course Sciences, Faculty of Life Sciences and Medicine, Kings College London, United Kingdom

## Abstract

This cross-sectional study investigates the association of the COVID-19 pandemic with rates of pediatric clinical trial publication.

## Introduction

During the COVID-19 pandemic, the research community responded rapidly to deliver highly collaborative trials covering a range of treatment and vaccine studies. However, such large-scale prioritization likely diverted resources, raising concerns about associated changes in other areas of research,^1^ including reduced initiation of clinical trials.^2^ Postulating that these changes would be associated with reduced trial progress and completion, we hypothesized a decrease in published clinical trials in children and young people since the pandemic.

## Methods

In this cross-sectional study, we conducted a PubMed search identifying pediatric publications, including randomized and other clinical trials, and further interrogated regional variations in publications (eMethods in Supplement 1). We used Medical Subject Headings (MeSH) to evaluate pediatric subspecialties. Publications per year were analyzed as a series with pandemic years (2020-2022) compared with the prepandemic mean (2018-2019). We also compared publication data with COVID-19 infections, deaths, and country populations.^3^ Guy’s and St Thomas’ National Health Service Foundation Trust determined that because no patient data were collected or analyzed, institutional review board approval was not required. The STROBE reporting guideline was followed when applicable.

## Results

**Figure 1.**
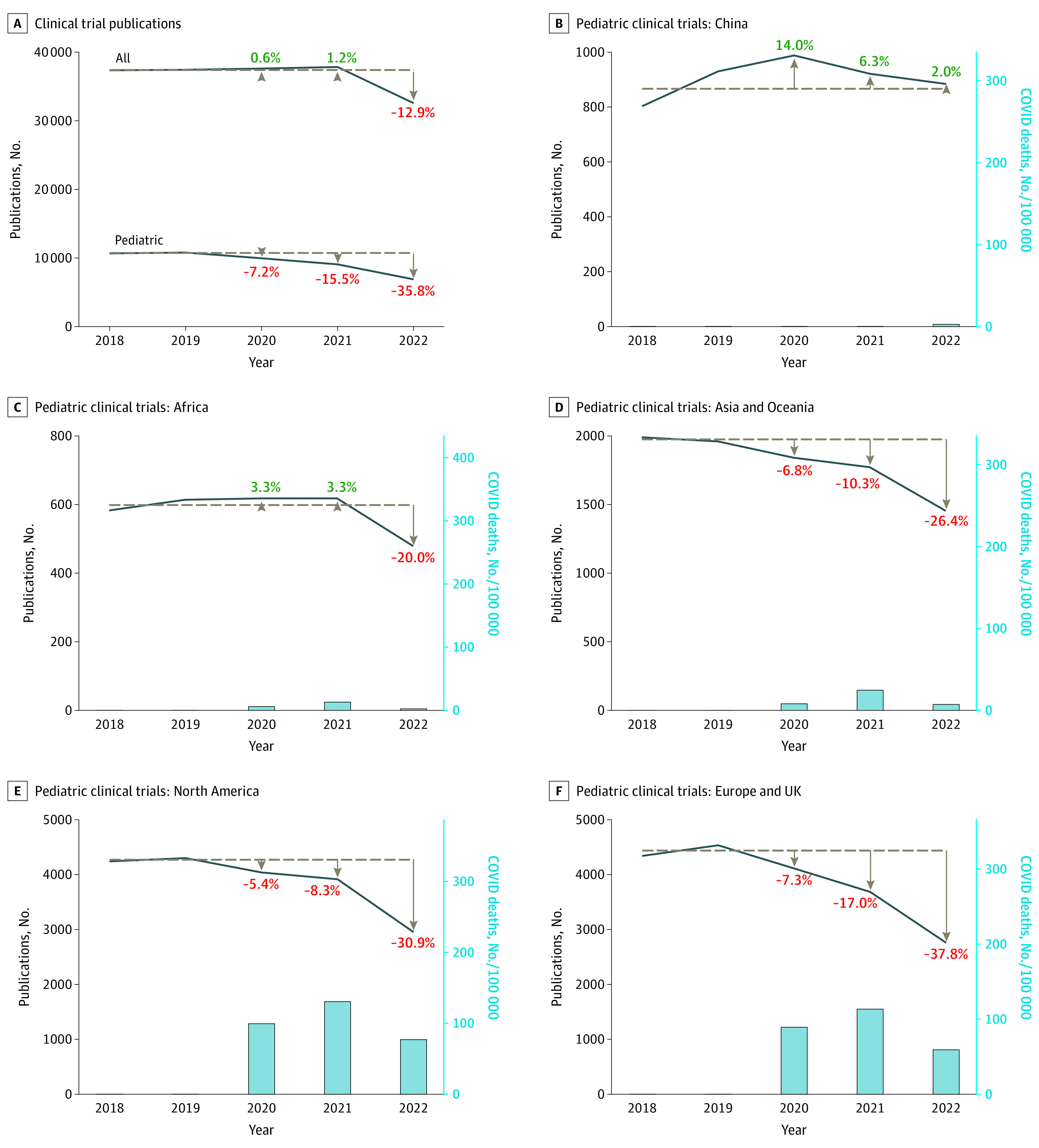
Published Clinical Trials Before and After the COVID-19 Pandemic Dashed lines indicate prepandemic (2018-2019) means in clinical trial publications. The first 2 panels show a greater decrease in published clinical trials in 2020 to 2022 in children compared with all trials. Subsequent panels show regional data ordered according to the magnitude of the decrease in published pediatric clinical trials in 2022; regions with greater COVID-19 burden during the pandemic (illustrated by COVID-19 death rates) had the greatest reductions in pediatric clinical trial publications.

**Figure 2.**
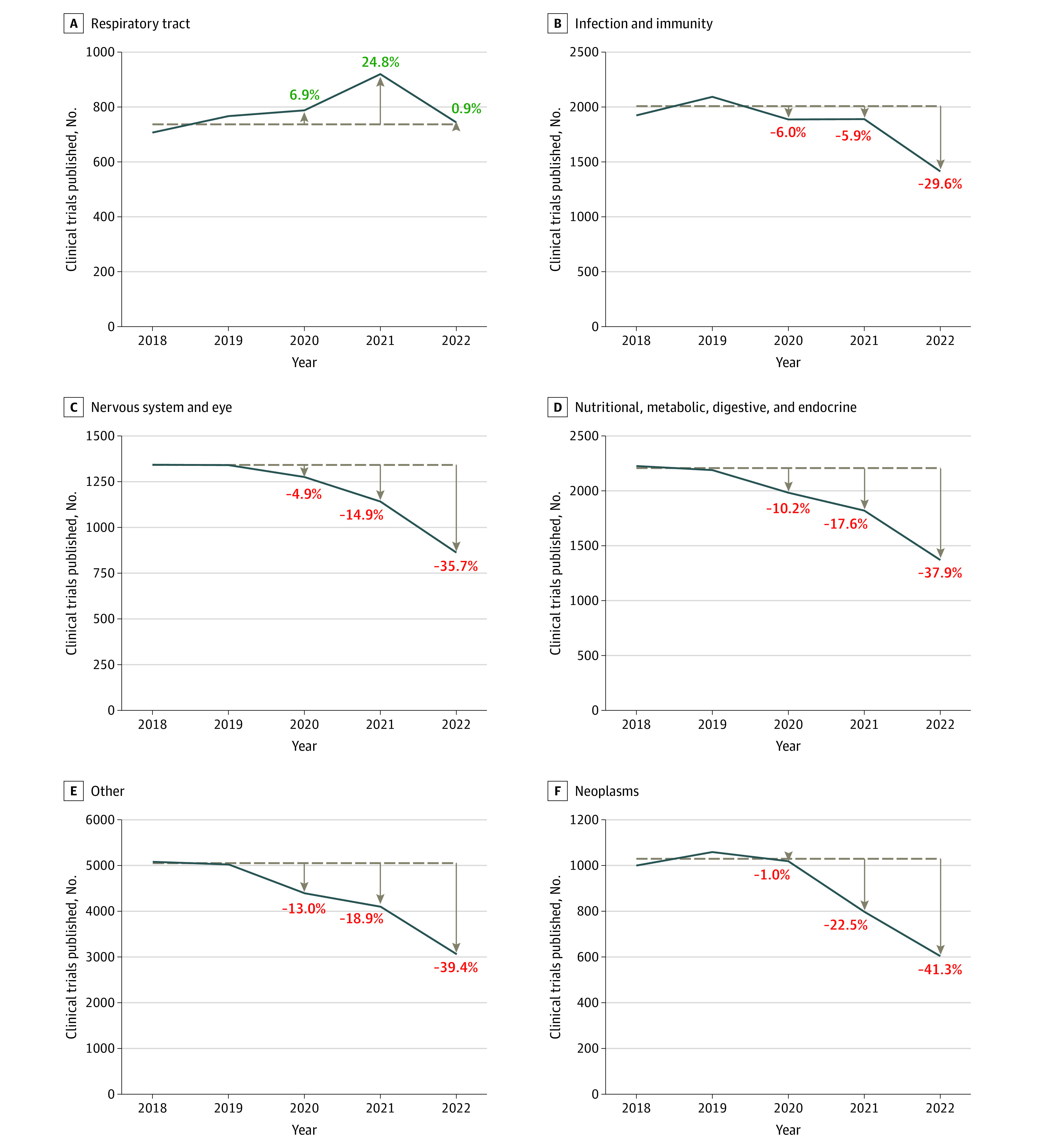
Research Outputs Across Pediatric Subspecialties Before and After the COVID-19 Pandemic Dashed lines indicate prepandemic (2018-2019) means in pediatric clinical trial publications by subspecialty. Publications in 2020 to 2022 were reduced across all pediatric subspecialties except respiratory disorders. Panels are ordered according to the magnitude of the decrease in 2022. Other conditions include urogenital diseases; cardiovascular diseases; wounds and injuries; congenital, hereditary, and neonatal diseases and abnormalities; skin and connective tissue diseases; musculoskeletal diseases; and otorhinolaryngologic disorders.

Despite an increase in pediatric publications during and after the pandemic (13% in 2020, 18.7% in 2021, and 8.9% in 2022 from a prepandemic mean of 222 700 publications), there was a year-on-year incremental reduction from 2020 to 2022 in published pediatric clinical trials (7.2% in 2020, 15.5% in 2021, and 35.8% in 2022 from prepandemic mean of 10 730 publications) (Figure 1) and randomized clinical trials (1.3% in 2020, 8.9% in 2021, and 25.7% in 2022 from a prepandemic mean of 7802 publications). This trend occurred across childhood conditions except respiratory disorders (Figure 2). Regions varied, with the greatest change in Europe and the UK (37.8% reduction in 2022), whereas China had a 2% increase in 2022 (Figure 1). Publication of all clinical trials decreased 12.9% in 2022.

## Discussion

Data from this cross-sectional study add to previous reports highlighting the association of the pandemic with non–COVID-19 trial publication^1,2^ by demonstrating a disproportionate reduction in pediatric clinical trial publications, with an almost 3-fold greater decrease in 2022 compared with all trials (35.8% vs 12.9%). We identified important regional variations, reflecting varying outcomes associated with COVID-19. Although we used death as a surrogate measure of COVID-19 burden, many factors contributed to the association of large numbers of COVID-19 infections with outcomes in health care resilience.^4^ National and local measures of workforce redeployment and social distancing were ultimately associated with stoppage or significant limitation of non-COVID-19–related research, as evidenced by the 69% reduction in initiation of non–COVID-19 clinical trials in the US.^2^ Disruption of health care systems may have also been associated with patient enrollment and retention in studies.

Globally, governments face major challenges to ameliorate the consequences of the pandemic. The US recovery program (updated in 2022) focused on reducing COVID-19 infection rates, minimizing impact on health services, and improving global health security.^5^ Nevertheless, worrying aspects of our data were the lack of recovery and an enlarging gap in research outputs, particularly in child and adolescent health. Clinical trials drive progress in medical care; hence, our findings are concerning. It is imperative that researchers, funders, regulators, and professional bodies work collaboratively to prevent further decline and use resources optimally by minimizing research waste. Additionally, lessons learned and innovations from COVID-19 research should be applied to the wider range of clinical trials. A 2022 publication from the Conect4Children expert advice group provided recommendations for increased flexibility of trial design, operational efficiency, and enhanced communication between stakeholders and regulatory bodies.^6^ Implementing these recommendations may optimize chances of successful completion.

The key limitation of this study was the use of publications as a proxy indicator of research activity. Further research is needed to determine if this constitutes genuine attrition in research activity or simply a lag to publication associated with COVID-19 disruptions to the arduous clinical trial process, which often takes years to plan, fund, and conduct. Elucidating specific causes, such as publication priority, study recruitment and participation, and study design, may help plan more focused ameliorating interventions.

## Notes

Supplement 1. **eMethods.** PubMed Search Strategy

Supplement 2.
Data Sharing Statement
