# Supplementary material for: Global Association of the COVID-19 Pandemic With Pediatric Clinical Trial Publication
Source: JAMA Netw Open. 2023 Jul 28;6(7):e2326313. doi: 10.1001/jamanetworkopen.2023.26313 (PMC10383004; doi:10.1001/jamanetworkopen.2023.26313)
Supplement: Supplement 1. — eMethods. PubMed Search Strategy [file jamanetwopen-e2326313-s001.pdf]

Supplemental Online Content

Grantham-Hill S, Eyre M, Ramanan AV, Modi N, de Wildt SN, Lim M. Global association of the COVID-19 pandemic with pediatric clinical trial publication. *JAMA Netw Open*. 2023;6(7):e2326313. doi:10.1001/jamanetworkopen.2023.26313

eMethods. PubMed Search Strategy

This supplemental material has been provided by the authors to give readers additional information about their work.

© 2023 Grantham-Hill S et al. *JAMA Network Open*.

eMethods. PubMed Search Strategy

To identify paediatric publications, we used the search terms: **Infant** OR **Infants** OR **Infancy** OR **Newborn** OR **Baby** OR **Babies** OR **Neonate** OR **Neonates** OR **Neonatal** OR **Child** OR **Children** OR **Childhood** OR **Toddler** OR **Toddlers** OR **Pediatric** OR **Pediatrics** OR **Paediatric** OR **Paediatrics** OR **Adolescent** OR **Adolescence.**

Clinical Trial and Randomised Controlled Trial filters were used to identify respective paediatric clinical trials and randomised controlled trials.

To identify all clinical trials published, we used the search term **Clinical Trials** and additionally using Clinical Trial and Randomised Control Trial filters.

To identify regional research output: -
North America: US OR USA OR United States OR America OR United States of America OR Canada.
Europe: Germany OR France OR Italy OR Spain OR Ukraine OR Poland OR Romania OR Netherlands OR Belgium OR Czech Republic OR Greece OR Portugal OR Sweden NB: Nations with >10m population/**UK**: UK OR England OR Wales OR Scotland OR Northern Ireland OR United Kingdom OR Britain OR British.
China: China OR People's Republic of China.
Asia: India OR Indonesia OR Pakistan OR Bangladesh OR Japan OR Philippines OR Vietnam OR Thailand OR Myanmar OR South Korea. NB Top 10 by population/**Oceania:** Australia **OR** New Zealand.
Africa: Nigeria OR Ethiopia OR Egypt OR Congo OR South Africa OR Tanzania OR Kenya OR Uganda OR Algeria OR Sudan. NB Top 10 by population.

To identify publication within subspecialties, we combined search with major topic MeSH diseases terms: respiratory tract diseases (C08); infections (C01); urogenital diseases (C12); endocrine system diseases (C19); eye diseases (C11); Congenital, hereditary and neonatal diseases and abnormalities (C16); nervous system diseases (C10); musculoskeletal diseases (C05); neoplasms (C04); digestive system diseases (C06); nutritional and metabolic diseases (C18); cardiovascular diseases (C14); immune system diseases (C20); skin and connective tissue diseases (C17); wound and injuries (C26); and otorhinolaryngologic disease (C09)

Subspecialties result were placed in 6 groups for analysis. Similar sub-specialties were grouped together and remaining were grouped as other.

1. Infection (CO1) and immune system disease(C20)
2. Respiratory tract (CO8)
3. Endocrine system disease(C19), nutritional and metabolic disease (C18) and digestive system disease(C06)
4. Neoplasm(C04)
5. Nervous system disease(C10) and eye disease(C11)
6. Other: Urogenital disease (C12); cardiovascular disease(C14); wound and injuries(C26); congenital, hereditary and neonatal diseases and abnormalities (C16); skin and connective tissue(C17); musculoskeletal disease(C05) and otorhinolaryngologic (C09)

© 2023 Grantham-Hill S et al. *JAMA Network Open*.
