# Supplementary material for: Global Association of the COVID-19 Pandemic With Pediatric Clinical Trial Publication
Source: JAMA Netw Open. 2023 Jul 28;6(7):e2326313. doi: 10.1001/jamanetworkopen.2023.26313 (PMC10383004; doi:10.1001/jamanetworkopen.2023.26313)
Supplement: Supplement 2. — Data Sharing Statement [file jamanetwopen-e2326313-s002.pdf]

# Data Sharing Statement

Grantham-Hill. Global Association of the COVID-19 Pandemic With Pediatric Clinical Trial Publication. *JAMA Netw Open*. Published July 28, 2023.
doi:10.1001/jamanetworkopen.2023.26313

**Data**
**Data available:** Yes
**Data types:** Data (not involving human participants)
**How to access data:** Direct request to corresponding author (ming.lim@gstt.nhs.uk)
**When available:** With publication

**Supporting Documents**
**Document types:** None

**Additional Information**
**Who can access the data:** Anyone requesting
**Types of analyses:** Any purpose
**Mechanisms of data availability:** Signed data access agreement
**Any additional restrictions:** No
